# Reducing Loneliness through the Power of Practicing Together: A Randomized Controlled Trial of Online Dyadic Socio-Emotional vs. Mindfulness-Based Training

**DOI:** 10.3390/ijerph21050570

**Published:** 2024-04-29

**Authors:** Hannah Matthaeus, Malvika Godara, Sarita Silveira, Martin Hecht, Manuel Voelkle, Tania Singer

**Affiliations:** 1 Social Neuroscience Lab, Max Planck Society, 10557 Berlin, Germany; hannah.matthaeus@social.mpg.de (H.M.); malvika.godara@social.mpg.de (M.G.);; 2 Department of Psychology, Humboldt-Universität zu Berlin, 12489 Berlin, Germany; manuel.voelkle@hu-berlin.de; 3 Institute of Medical Psychology, Charité—Universitätsmedizin Berlin, 10117 Berlin, Germany; 4 Faculty of Humanities and Social Sciences, Helmut Schmidt University, 22043 Hamburg, Germany; martin.hecht@hsu-hh.de

**Keywords:** mental training, social connectedness, app-delivered intervention, randomized controlled trial, mental health

## Abstract

Loneliness has become a pressing topic, especially among young adults and during the COVID-19 pandemic. In a randomized controlled trial with 253 healthy adults, we evaluated the differential efficacy of two 10-week app-delivered mental training programs: one based on classic mindfulness and one on an innovative partner-based socio-emotional practice (Affect Dyad). We show that the partner-based training resulted in greater reductions in loneliness than the mindfulness-based training. This effect was shown on three measures of loneliness: general loneliness assessed with the 20-item UCLA Loneliness Scale, state loneliness queried over an 8-day ecological momentary assessment in participants’ daily lives, and loneliness ratings required before and after daily practice. Our study provides evidence for the higher efficacy of a mental training approach based on a 12 min practice conducted with a partner in reducing loneliness and provides a novel, scalable online approach to reduce the increasing problem of loneliness in society.

## 1. Introduction

Loneliness has been defined as the feeling of deficiencies in the frequency and quality of social contact [1]. Research has shown that subjectively experienced loneliness is relevant for predicting objective outcomes, such as increased mortality [2], cardiovascular diseases [3], and cognitive functioning [4]. Higher loneliness has also been linked to an increased risk of depression, anxiety, and suicidal ideation [5]. While research findings have been mixed regarding the relationship between age and loneliness [6,7,8], recent studies have consistently noted a trend toward increased loneliness among young adults [9,10], with younger individuals often reporting the highest levels of loneliness [11,12]. This rising trend in loneliness, also referred to as a loneliness epidemic [13], has been amplified in recent years by the COVID-19 pandemic [14]. In Germany, loneliness increased during the first lockdown, particularly affecting young adults [15,16]. These alarming developments call for effective measures and intervention programs to mitigate loneliness on a large scale, with a focus on digital approaches that are scalable and easily accessible, even in times and under conditions that require staying at home.

Several approaches for the reduction of loneliness have been developed to address the growing issue of loneliness, with meditation, mindfulness, and social cognitive training rated as particularly promising solutions [17]. The field of contemplative science has gained prominence in recent years for improving mental health and well-being [18,19] as well as social connectedness [20]. Previous research has shown that classic 8-week mindfulness programs such as mindfulness-based stress reduction (MBSR) [21] and more compassion-based approaches can reduce loneliness [22]. While a review from Veronese et al. (2021) [17] reports successful loneliness reduction through mindfulness, a closer look at the studies referenced reveals that most interventions were conducted in person. These in-person trainings typically involved weekly sessions led by a teacher [23]. However, research on training based on online applications is limited. One study, which utilized a two-week smartphone-based training program focusing on daily attention and acceptance practices, reported reductions in state loneliness as assessed with ecological momentary assessment (EMA) but not in trait measures of loneliness [24]. There remains a lack of research on the efficacy of low-dose mindfulness or compassion-based mental training delivered online and via mobile apps, despite evidence suggesting that digital interventions can be beneficial for combating loneliness, specifically among non-elderly adults [25].

In addition to the specific content and skills a practice focuses on, mental training programs also differ in the modality in which they are practiced. Although most contemplative practices are performed alone due to their origin in classic meditation, there is a growing interest in intersubjective, dyadic approaches such as inquiry methods [26] or other intersubjective practice formats [27]. In the ReSource project [28], new contemplative partner-based practices called Contemplative Dyads were introduced. They involve a structured dialogue where two randomly assigned partners take turns answering and exploring specific questions while the other partner is empathically listening without interrupting. These interactions were found to increase social connectedness and social disclosure over a 3-month period of practice [20]. Research has shown that social connectedness can act as a buffer against loneliness [29] and can be increased through both intrapersonal interventions based on compassion [30] and socio-emotional partner-based dyadic training [20]. This suggests that the novel types of daily Dyads, which emphasize social connections with a partner, may serve as an auspicious approach to reducing subjective loneliness. However, in the ReSource project, which was an extensive in-person large-scale study that included 3-day in-person retreats and weekly in-person sessions with teachers, both types of Dyads (Affect Dyad and Perspective Dyad), introduced as core practices, were always combined with more classic meditation practices [28]. Studies have yet to explore the isolated effects of socio-emotional dyadic practice (Affect Dyad) on reducing loneliness and enhancing social connectedness, especially its differential efficacy in comparison to classic mindfulness practices and in app-delivered formats.

To address these gaps, we compared the efficacy of a purely online partner-based socio-emotional training (Affect Dyad) with classic mindfulness training performed over 10 weeks with weekly online sessions with teachers and daily 12 min practice in reducing loneliness using a multi-method approach. To assess different aspects of loneliness, we employed (1) a validated trait scale, the UCLA Loneliness Scale [31], (2) an 8-day EMA of loneliness dynamics assessed in participants’ daily lives, and (3) a daily state measure of perceived loneliness assessed directly before and after each daily practice session over the 10 weeks of training.

Given the social nature of the novel partner-based practice, we expected the dyadic training to be more effective in increasing social connectedness and reducing loneliness than a comparable mindfulness practice performed alone. This expectation stems from the fact that the dyadic practice involves more self-disclosure, which is known to be associated with decreasing loneliness [32]. Additionally, we aimed to explore potential factors that could drive changes in loneliness in the two different interventions. Previous studies have identified common humanity [33], social contacts [24], social support [34], a sense of belonging [35], and a low fear of compassion [36] as mediators of loneliness. However, these factors have not been directly compared in the context of different contemplative practices and were therefore assessed in this study.

## 2. Materials and Methods

The data reported in this study were gathered as part of the CovSocial project, a longitudinal mental-health study initiated during the early weeks of the first lockdown in Germany due to the COVID-19 pandemic (for the study protocol, see [37]). The main objectives of the project were to investigate changes in psychological vulnerability, resilience, and social cohesion resulting from a crisis such as the COVID-19 pandemic (phase 1) and to examine the differential effects of online mental training programs in a randomized controlled trial (RCT; phase 2; Trial Registration: ClinicalTrials.gov NCT04889508 on 17 May 2020).

### 2.1. Sample

**Figure 1:**
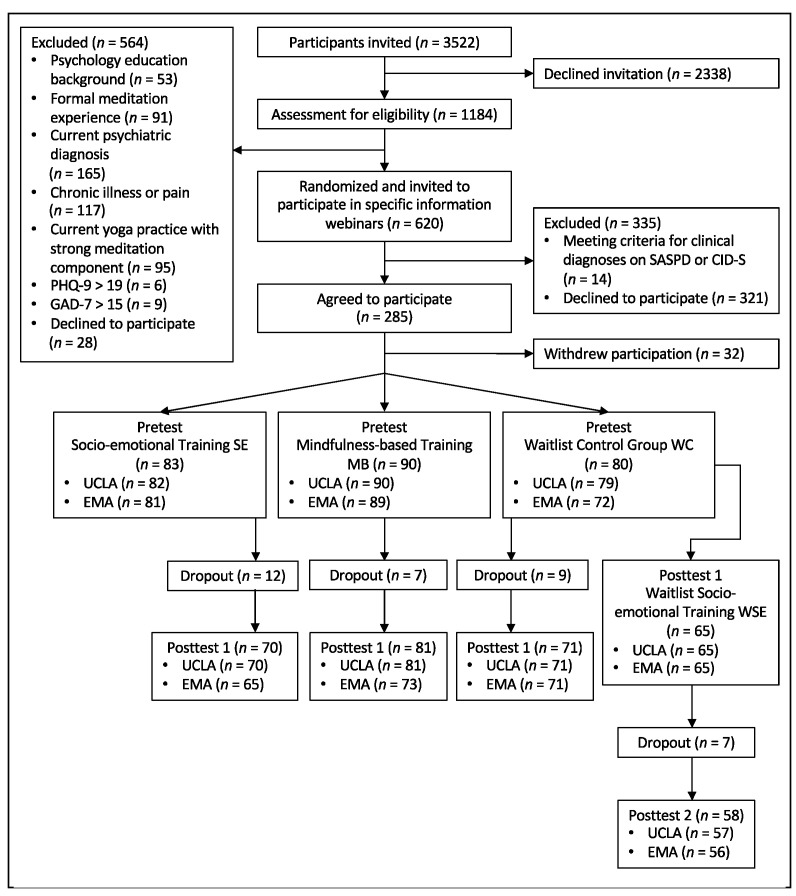
CONSORT flow diagram. EMA = ecological momentary assessment; UCLA = University of California Loneliness Scale; SASPD = standardized assessment of severity of personality disorder; CID-S = composite international diagnostic screener; GAD-7 = Generalized Anxiety Disorder-7; PHQ-9 = Patient Health Questionnaire-9.

The CovSocial project’s phase 2 recruited participants from a community sample in Berlin, initially selected from phase 1 participants (Figure 1). Various recruitment methods, including registration office sampling and social media advertising, led to 7214 registrations, with 3522 completing the phase 1 questionnaires. These 3522 individuals were invited to a pre-screening for phase 2, in which eligibility based on specific criteria was assessed. Phase 1 inclusion criteria comprised age (18–65), Berlin residency, and German proficiency. Participants were excluded from phase 2 if they lacked internet access or necessary technical equipment, had a background in psychology, engaged in regular spiritual practices, took specific medications, participated in stress reduction programs, suffered from chronic illness or pain, had a psychiatric history, or exceeded cutoff scores on questionnaires assessing alexithymia [38], depression [39], and anxiety [40], including an item for suicidality. Eligible participants were randomly assigned to three groups, initially oversampling for ideal group sizes. Detailed information about the recruitment process, study design, and interventions can be found in Supplementary Material S1 and the study protocol [37].

**Figure 2:**
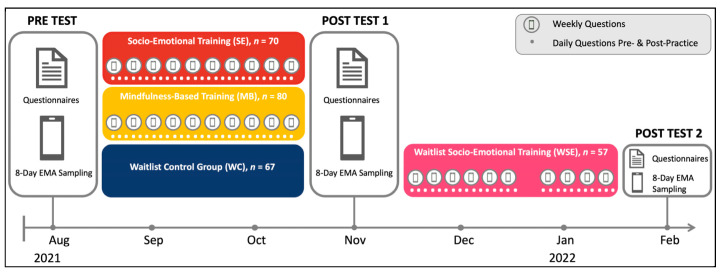
Study design of the CovSocial project phase 2, including study measures for loneliness assessment.

A total of 253 participants (age: M = 44.36, SD = 11.48; 75.5% female) participated in the pretest of phase 2. After 10 weeks of treatment in the two intervention groups, we assessed measures in posttest 1 from 70 participants of the socio-emotional training (SE), 81 of the mindfulness-based training (MB), and 71 of the Waitlist Control Group (WC). In addition, 65 participants in WC continued after posttest 1, undergoing socio-emotional training in a second intervention phase as well as assessments at posttest 2 (WSE). Figure 2 depicts the study design, including the measurements relevant to the reported analyses.

This study was preregistered at the Open Science Framework (https://osf.io/3nsjc, accessed on 23 April 2024; see Supplementary Material S3) and is in accordance with the Declaration of Helsinki. Ethical approval was received by the institutional review board of Charité—Universitätsmedizin Berlin (#EA4/081/21). All participants provided written informed consent and were reimbursed for their time spent on testing at the rate of EUR 10 per hour. Table 1 displays sample descriptives for this study.

### 2.2. Procedure

All participants in SE, MB, and WSE underwent a 10-week training program with a 12 min daily app-based practice six times per week and a weekly 2 h online coaching session in smaller groups of 15 to 20 participants, supported by four expert mindfulness and Dyad teachers randomly assigned to these subgroups. The training differed between MB and SE/WSE.

In MB, the CovSocial app provided guided meditation recordings on breathing, listening, and open awareness (see presence module [28]).

In SE and WSE, the participants engaged in mental training through contemplative Dyads, which are structured meditations performed with a partner [20]. During this training, they were paired with a randomly assigned partner from their group, with the partners changing after each weekly coaching session. During each practice, one partner began the session by talking for 2.5 min about a situation from the last 24 h in which they experienced a difficult emotion and how they felt this emotion in their body, followed by talking for 2.5 min about a gratitude-eliciting situation and related bodily experiences. The listener was instructed to remain silent and listen empathically without judgment, both externally and internally, while resonating with the feelings of the other person. After those 5 minutes, the roles of speaker and listener switched, and the partner that was listening in the first half of the practice got to share their respective difficult and grateful situations with the related bodily sensations. Before and after each practice, participants of all groups answered questions about their subjective state at that moment. A detailed description of the intervention protocol, including the onboarding procedure and training, can be found in Supplementary Material S1.

### 2.3. Measures

This study presents data on loneliness and social connectedness, as well as compliance and motivation for the two types of mental training programs offered in the CovSocial phase 2 study. The number of completed practices measured compliance during the intervention period and during the voluntary continuation of practice for 10 weeks after each posttest. Motivation was assessed before each practice using a rating scale from 0 (“not at all”) to 4 (“very much”).

The primary outcome of loneliness was evaluated at pretest, posttest 1, and posttest 2 using two different methods. The first was a validated psychometric questionnaire, the 20-item UCLA Loneliness Scale (UCLA-20; score range, 1–5; [31,41]), with a higher score indicating greater loneliness. The second method was an 8-day ecological momentary assessment (EMA), in which participants were asked to rate their level of loneliness on a scale of 0–8 when receiving push notifications on their mobile devices every three hours from the time of awakening until 9 pm. Additionally, loneliness was assessed daily with a self-generated item (rating scale 0–8; “How lonely do you feel right now?”), immediately before and after each daily practice during the 10-week intervention program. Social closeness with the respective Dyad partner was measured only in Dyad groups (SE, WSE) using the Inclusion of Other Scale (IOS; [42]). Further, the extent of personal self-disclosure during each Dyad was assessed on a rating scale from 0 (“not at all”) to 4 (“very personal”).

In addition, variables that might be considered mediating factors of change in loneliness were assessed once a week during the two intervention periods in a pseudorandomized design. Using one item each, these measures included common humanity (State Self-Compassion Scale (SSCS) [43]; “I reminded myself that there are many other people in the world who feel as I do”), frequency and valence of social contacts (self-generated, “How often did you have social contact during the past week?” and “On average, how pleasant were these social contacts?”), social support (Brief-COPE [44]; “I have sought help and support from others”), belonging to friends and the world (Inclusion of Other in the Self Scale [42]; “Draw the circles to best represent your affiliation with the following group: Me and Friends, Me and World Population”), and fear of compassion (FoC [45]; “I do not want to be compassionate with myself because it might make me soft and easy to take advantage of”, “I do not want to be compassionate with myself because I do not want to become dependent on it”).

### 2.4. Statistical Analyses

Descriptive statistics for UCLA and EMA ratings and the internal consistency of UCLA are reported in Supplementary Tables S1 and S2. Power analyses were conducted using G*Power [46] for analysis of variance with repeated measurements and interactions between group and within-group variables using elements: *α* = 0.05, power (1- β) = 0.80, 3 groups, 2 measurement occasions, *f* = 0.10, and *r* = 0.39 for repeated measurements (see Supplementary Material S2 for further details). All outcome variables were standardized by their overall standard deviation (pooled across time points and groups) to ensure comparability across the different measurement time points and groups. Participants’ sex and age were included as covariates in all models. Analyses of change in primary outcomes used linear mixed-effects models with fixed effects for the intervention group and time (pretest, posttest 1, posttest 2), an interaction between group and time, and individual-level random intercepts. The WC group was used as the reference group, with backward difference coding for the time factor. Planned contrasts considered the effect of treatment in SE, MB, and WSE as contrasted against the WC group. We report standardized estimates and *p*-values. Model estimates of planned contrasts reflect effect size estimates classified as small (≥0.20), medium (≥0.50), or large (≥0.80). Data are analyzed using the intention-to-treat approach, ensuring that all participants who provided data for at least the pretest timepoint are included in the analysis for each outcome.

Changes in daily measured variables before and after daily practice in SE and MB were analyzed using linear mixed-effects models with fixed effects for group, day, and measurement occasion (dummy coded variable for whether the measurement occasion was before or after the practice; reference group: pre-practice scores) as well as their two- and three-way interactions, individual-level random intercepts, and correlated random slopes for time and measurement occasion with SE as the reference group. A second model included WSE with fixed effects for time, measurement occasion, and their interaction, as well as individual-level random intercepts and correlated random slopes. For variables assessed either before (i.e., motivation) or after (i.e., self-disclosure) daily practice, models included fixed effects for group, day, their interaction, and individual-level random intercepts and slopes.

Planned contrasts included change over time (day and measurement occasion) for each group separately (SE, MB, and WSE) and for differential effects between SE and MB. Preregistered hypotheses were tested one-sidedly. Outcome analyses were conducted using the lme4 [47] and multcomp packages [48] in R (version 4.0.2; [49]). Change slopes of weekly assessed mediator variables were extracted in the form of estimated fixed effects of time from linear mixed-effects models that included random intercepts and slopes. Mediation models were conducted using the lavaan package [50], with the pre-to-posttest change in each outcome variable defined as the dependent variable, group as an independent variable, and extracted slopes as mediators of change. Since mediator variables were not assessed in WC, the mediation models used a dummy-coded group variable with MB defined as the reference group. An effect was deemed significant if zero was not included in the 95% confidence interval. Bootstrap confidence intervals will be reported using 5000 bootstrap iterations in each model.

## 3. Results

### 3.1. Engagement

**Figure 3:**
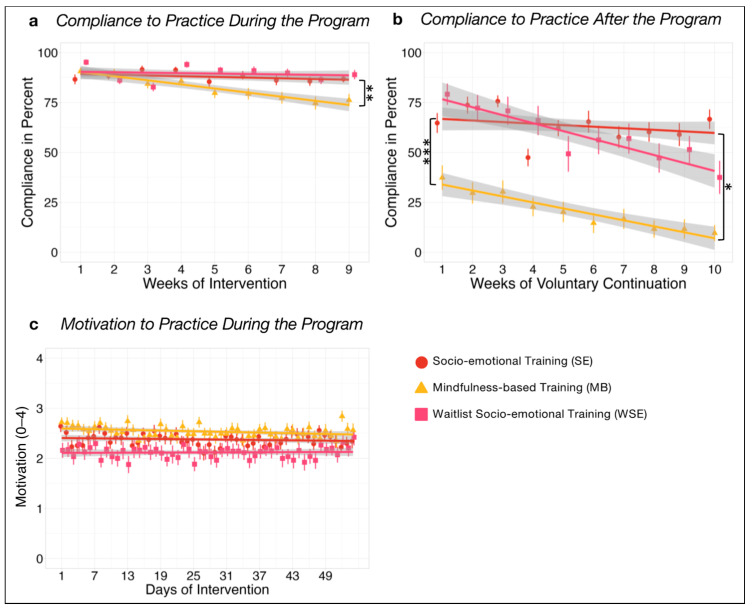
Time courses with group mean and standard errors of (**a**) compliance of training during the intervention period, (**b**) compliance of voluntary continuation for another 10 weeks of training after the posttest with the study app, and (**c**) self-rated motivation of training during the intervention period. Bonferroni corrected significance level of * α = 0.05, ** α = 0.01, and *** α = 0.001.

Compliance to practice (Figure 3) showed no significant change over time in SE (*β*_SE_ = −0.02, *p* = 1) but decreased significantly in MB (*β*_MB_ = −0.10, *p* < 0.001), with a significant difference in change over time between the groups (*β*_diff_ = −0.08, *p* = 0.001). Baseline levels at week 1 showed no significant difference between SE and MB (*β_diff_* = 0.15, *p* = 1). In WSE, compliance showed no significant change over time (*β*_WSE_ = −0.01, *p* = 0.435).

Voluntary continuation of practice (Figure 3) for a further 10 weeks after the posttest showed no significant change over time in SE (*β*_SE_ = −0.02, *p* = 0.889). In MB, the decrease was significant (*β*_MB_ = −0.08, *p* < 0.001), with a significant difference between groups (*β*_diff_ = −0.06, *p* = 0.027). A significant difference in baseline levels at week 1 reflected a significantly higher continuation attendance in SE compared to MB (*β*_diff_ = −0.82, *p* < 0.001). In WSE, continuation compliance significantly decreased over time (*β*_WSE_ = −0.11, *p* < 0.001).

No significant change in motivation (Figure 3) was found over time in SE (β_SE_ = −0.01, *p* = 1), MB (β_MB_ = −0.05, *p* = 0.055), or WSE over time (β_WSE_ = 0.00, *p* = 0.985), and motivation did not differ between SE and MB in week 1 (β_diff_ = −0.13, *p* = 0.747).

### 3.2. Primary Outcome

**Figure 4:**
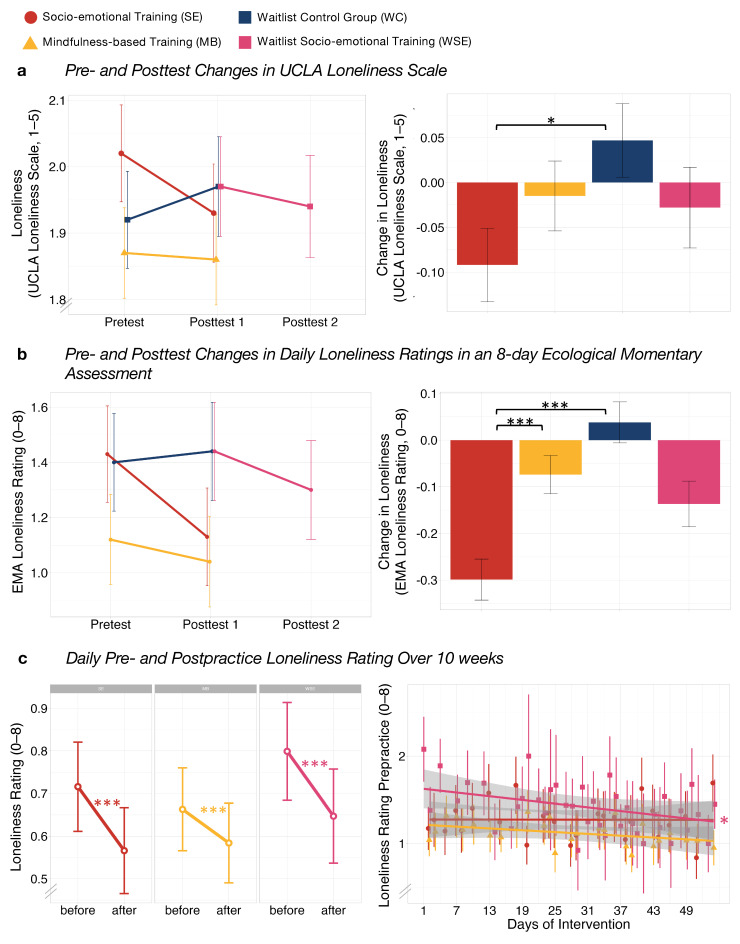
Group differences at pretest, posttest 1 and posttest 2, and of pre- to posttest changes using unstandardized estimates of the linear mixed model of (**a**) UCLA-20 and (**b**) ecological momentary assessment (EMA). Group differences in (**c**) mean daily pre- to post-practice changes in loneliness ratings and trajectories over 10 weeks of training in daily pre-practice loneliness ratings. Difference Scores are extracted from linear mixed models with sex and age as covariates. Data are presented as means and standard errors. Bonferroni corrected significance level of * α = 0.05 and *** α = 0.001.

A significant decrease in UCLA-20 scores (Figure 4) was observed for SE compared to WC (*β*_SE_ = −0.23, *p* = 0.035) but not to MB (*β*_diff_ = −0.13, *p* = 0.343), indicating a small effect size for the reduction in loneliness within the SE group. MB vs. WC (*β*_MB_ = −0.10, *p* = 0.551), as well as WSE vs. WC (*β*_WSE_ = −0.12, *p* = 0.619), showed no significant change.

Loneliness ratings measured with an EMA design (Figure 4) showed a significant training-related reduction for SE compared to WC (*β*_SE_ = −0.19, *p* < 0.001) and MB (*β*_diff_ = −0.13, *p* < 0.001), with small effect sizes observed for both comparisons. No significant change was found for MB compared to WC (*β*_MB_ = −0.07, *p* = 0.123) and WSE compared to WC (*β*_WSE_ = −0.10, *p* = 0.055).

A significantly greater decrease in change in loneliness from before to after each daily practice (Figure 4), i.e., a direct practice effect, was observed for SE (*β*_SE_ = −0.15, *p* < 0.001) and MB (*β*_MB_ = −0.08, *p* < 0.001), indicating small effect sizes for both groups. Differences between groups were not significant (*β*_diff_ = 0.07, *p* = 0.055), with a trend indicating a possibly greater direct decrease in loneliness for SE. No change over time was found for daily pre-practice loneliness in SE (*β*_SE_ = −0.00, *p* = 1) or MB (*β*_MB_ = −0.03, *p* = 0.414). WSE showed a significant decrease over time (*β*_WSE_ = −0.05, *p* = 0.035) and a significant direct practice effect (*β*_WSE_ = −0.15, *p* < 0.001), with small effect sizes observed for both findings.

**Figure 5:**
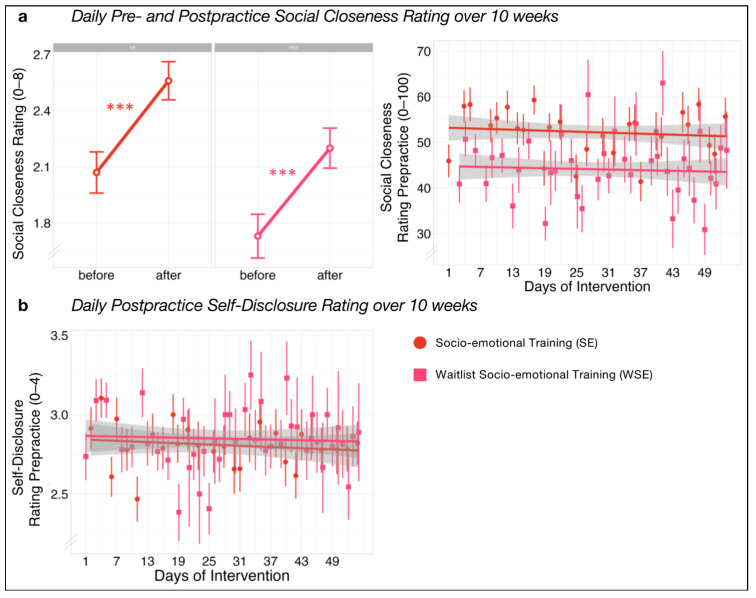
Group differences in (**a**) mean daily pre- to post-practice changes in social closeness ratings and trajectories over 10 weeks of training in daily pre-practice social closeness ratings. Group differences in trajectories over 10 weeks of training in daily post-practice self-disclosure ratings (**b**). Difference Scores are extracted from linear mixed models with sex and age as covariates. Data are presented as means and standard errors. Bonferroni corrected significance level of *** α = 0.001.

Social closeness ratings showed a significant increase after each practice compared to before in both socio-emotional training groups, SE (*β*_SE_ = 0.48, *p* < 0.001) and WSE (*β*_WSE_ = 0.47, *p* < 0.001; Figure 5). No significant change over time (Figure 5) was observed in SE (*β*_SE_ = −0.01, *p* = 0.651) or WSE (*β*_WSE_ = −0.00, *p* = 0.854). Ratings of self-disclosure after each practice showed no change over time in either socio-emotional training groups (Figure 5), SE (*β*_SE_ = −0.01, *p* = 0.603), or WSE (*β*_WSE_ = −0.02, *p* = 0.695).

### 3.3. Potential Mediator Factors of Change

Potential mediators of change in loneliness were assessed by change over the intervention in weekly measured variables of common humanity, frequency and valence of social contacts, social support, sense of belonging to friends and the world, and fear of compassion. Fear of compassion (*β* = −0.07, *p* < 0.001) and sense of belonging to friends (*β* = −0.92, *p* = 0.008) decreased significantly over time in all groups (SE, MB, and WSE). No significant change was found for common humanity (*β* = 0.00, *p* = 0.919), frequency of social contacts (*β* = −0.03, *p* = 0.177), valence of social contacts (*β* = 0.03, *p* = 0.340), social support (*β* = −0.01, *p* = 0.682), and sense of belonging to the world (*β* = −0.50, *p* = 0.190).

Mediation analyses did not reveal any indirect significant effects of the intervention group on changes in primary outcomes via changes in common humanity, frequency and valence of social contacts, social support, sense of belonging to friends and the world, and fear of compassion (see Tables S3 and S4 in the Supplementary Materials). No mediation effects were found for changes in UCLA-20 and loneliness ratings of EMA from pretest to posttest 1 in SE and MB and from posttest 1 to posttest 2 for WSE.

## 4. Discussion

The present RCT aimed at investigating the differential efficacy of two 10-week app-delivered mental training—a classic mindfulness-based intervention and a novel partner-based socio-emotional training (Affect Dyad), both involving 12 min daily practice—on different outcomes of loneliness and social connectedness.

First, we compared motivation, compliance, and voluntary continuation between the two types of mental training. Participants in the dyadic socio-emotional training groups (SE and WSE) showed higher compliance and voluntary continuation after the official training program compared to the mindfulness-based intervention (MB), despite both intervention groups reporting similar levels of motivation to perform the daily practice. The higher compliance levels observed in the socio-emotional training, requiring daily scheduled joint sessions throughout both the 10-week main program and the 10-week voluntary continuation period, may be due to the inherent accountability fostered by their shared commitments. In contrast to solitary practices, where skipping a session is easier, the social expectation inherent in dyadic training fosters a sense of obligation, hence translating individual motivation into tangible behavioral outcomes. This adherence to social norms likely contributes to increased attendance and engagement sustainability with daily mental training sessions, speaking for the sustainability of this novel dyadic format.

Second, we aimed to investigate the differential efficacy of the two online interventions on different markers of subjective loneliness. We found evidence that both groups of socio-emotional dyadic training (SE and WSE) led to greater reductions in dynamic state loneliness (EMA) compared to the mindfulness training group and waitlist control group. Similarly, we also observed greater reductions in general trait-level loneliness (UCLA) for the Affect Dyad training group as compared to the control group. However, this effect could only be observed in the SE group and not in the WSE group. Concerning daily ratings of loneliness immediately before and after practice, we found, on average, a decrease in loneliness in all three intervention groups. However, we could clearly observe a trend towards higher loneliness reduction in both Dyad groups as compared to the mindfulness group. Loneliness ratings before daily practice did not change over the 10-week intervention period for SE and MB but significantly decreased in WSE.

The reduction of loneliness after daily practice in both groups is in line with previous studies showing mindfulness- or compassion-based practices, typically performed alone, to be effective in reducing loneliness [17]. Thus, for example, a previous study on daily online meditation practice focusing on mentalizing and attention has demonstrated effectiveness in reducing state loneliness. Importantly, however, we extend these findings by showing, as predicted, that a novel partner-based practice, the Affect Dyad, outperforms mindfulness-based practice on all primary outcome measures in significantly decreasing loneliness in participants’ daily lives (EMA), as well as on the UCLA trait measure. In contrast, findings suggest that mindfulness practice, when applied as low-dose online training only, was not able to significantly reduce loneliness on both of these primary outcome measures.

Overall, we observed slightly stronger effects for the first Dyad cohort as compared to participants of the waitlist control group, who were administered active mental training in late winter. Multiple reasons could explain this finding. Firstly, the waitlist control group had a drop-out for the continuation of the training program and, therefore, had a lower sample size (*n* = 65) and, thus, less statistical power to detect an effect [51]. Additionally, seasonal effects might have influenced the efficacy of Affect Dyad training in WSE conducted in late winter 2021 during increasing numbers of COVID-19 infections, while SE received their training in early autumn.

The third aim of the present work was to assess improvements in social connectedness resulting from dyadic practice. Indeed, in line with previous findings [20], we observed an immediate increase in perceived social closeness after each daily Dyad. In contrast to previous findings, however, neither aspect of social connectedness showed improvement over the 10-week training period. It is possible that the smaller range of the scale utilized in the current study may have been insufficient to capture the subtle shifts in self-disclosure levels. Additionally, the absence of in-person meetings among participants may have contributed to a higher baseline level of self-disclosure due to anonymity [52]. On the contrary, the lack of replicable increase in social closeness over the course of the intervention might be due to the anonymity. These results suggest that while in-person meetings might facilitate the generalization of feelings of social connectedness during training, they could potentially lead to lower baseline levels of self-disclosure. Future research on Affect Dyad training will have to investigate these effects further.

Finally, we explored potential factors that may mediate intervention-related changes in loneliness observed before and after the 10-week training. No significant mediation effects could be observed. This may be due to the explorative nature of assessing potential mediators through one-item scales, which might lack sufficient reliability over time. Future studies will have to investigate possible mechanisms of change in loneliness using task-based measures and a more comprehensive assessment of the potential mediator variables.

## 5. Conclusions

Given the global rise of subjective loneliness in recent years, particularly amplified by the COVID-19 pandemic, many people, especially among younger demographics, experience loneliness and deficits in social connectedness. We found that purely online low-dose partner-based socio-emotional training was more effective than a comparable classic mindfulness-based program in reducing different aspects of state and trait-related loneliness. Furthermore, the dyadic practice seemed to result in higher compliance during and after the training, suggesting its potential for sustainable practice with enduring effects. Thus, we conclude that these daily dyadic intersubjective mental pieces of training provide a potentially scalable, low-cost digital approach to counter the threat of rising loneliness and its associated burdens, including severe mental health problems. Further, such socio-emotional partner-based pieces of training are even more promising as they come with additional benefits in reducing other aspects of mental health like depression and anxiety while at the same time boosting social skills such as empathy and (self-)compassion as well as resilience.

## Figures and Tables

**Table 1:** Characteristics of Participants at Pretest Split by Intervention Group (*n* = 253).

	Socio-Emotional Training (*n* = 83)	Mindfulness-Based Training (*n* = 90)	Waitlist Control Training (*n* = 80)
Age in years, mean ± SD	43.14 ± 11.80	44.14 ± 11.44	45.86 ± 11.15
Females, *n* (%)	65 (78.3%)	64 (71.1%)	62 (77.5%)
Married or cohabiting, *n* (%)	27 (32.5%)	32 (35.6%)	32 (40%)
Background of migration to current country of residence, *n* (%)	4 (4.8%)	10 (11.1%)	3 (3.8%)
Years of education, mean ± SD	18.49 ± 3.97	17.06 ± 3.52	18.41 ± 3.20
Employed full-time, *n* (%)	42 (50.6%)	57 (63.3%)	46 (57.5%)
Income > Berlin average monthly net (EUR 2175) ^a^, *n* (%)	52 (62.7%)	61 (67.8%)	56 (70.0%)
Lifetime prevalence of mental disorder, *n* (%)	17 (20.5%)	16 (17.8%)	18 (22.5%)

Note: ^a^ Amt für Statistik, Berlin-Brandenburg, 2019; https://www.statistik-berlin-brandenburg.de/publikationen (accessed on 23 April 2024).

## Data Availability

Data and R code are available from the corresponding author to other researchers on reasonable request.

## Supplementary Materials

The following supporting information can be downloaded at: https://www.mdpi.com/article/10.3390/ijerph21050570/s1, Supplementary Material S1: Mental Training Intervention Protocol CovSocial Phase 2: Summary; Supplementary Material S2: Power Analysis; Supplementary Material S3: Deviations from preregistration; Table S1: Descriptive statistics for loneliness (UCLA Loneliness Scale) and loneliness ratings measured with EMA, split by measurement occasion and group; Table S2: Cronbach’s alpha values for UCLA Loneliness Scale; Table S3: Mediation effects of slopes on change in outcome variables in the (waitlist) socio-emotional (SE and WSE) mental training compared to mindfulness-based mental training (MB); Table S4: Mediation effects of slopes on change in outcome variables in the socio-emotional (SE) mental training compared to waitlist socio-emotional (WSE) mental training.

## References

[1] Perlman D., Peplau L. (1981). Toward a social psychology of loneliness. Pers. Relatsh. Disord..

[2] Holt-Lunstad J., Smith T.B., Baker M., Harris T., Stephenson D. (2015). Loneliness and Social Isolation as Risk Factors for Mortality: A Meta-Analytic Review. Perspect. Psychol. Sci..

[3] Valtorta N.K., Kanaan M., Gilbody S., Ronzi S., Hanratty B. (2016). Loneliness and social isolation as risk factors for coronary heart disease and stroke: Systematic review and meta-analysis of longitudinal observational studies. Heart.

[4] Lara E., Martín-María N., De la Torre-Luque A., Koyanagi A., Vancampfort D., Izquierdo A., Miret M. (2019). Does loneliness contribute to mild cognitive impairment and dementia? A systematic review and meta-analysis of longitudinal studies. Ageing Res. Rev..

[5] Beutel M.E., Klein E.M., Brähler E., Reiner I., Jünger C., Michal M., Wiltink J., Wild P.S., Münzel T., Lackner K.J. (2017). Loneliness in the general population: Prevalence, determinants and relations to mental health. BMC Psychiatry.

[6] Clark D.M.T., Loxton N.J., Tobin S.J. (2015). Declining loneliness over time: Evidence from American colleges and high schools. Personal. Soc. Psychol. Bull..

[7] Mund M., Freuding M.M., Möbius K., Horn N., Neyer F.J. (2020). The stability and change of loneliness across the life span: A meta-analysis of longitudinal studies. Personal. Soc. Psychol. Rev..

[8] Xin S., Xin Z. (2016). Birth cohort changes in Chinese college students’ loneliness and social support: One up, as another down. Int. J. Behav. Dev..

[9] Buecker S., Mund M., Chwastek S., Sostmann M., Luhmann M. (2021). Is loneliness in emerging adults increasing over time? A preregistered cross-temporal meta-analysis and systematic review. Psychol. Bull..

[10] Twenge J.M., Spitzberg B.H., Campbell W.K. (2019). Less in-person social interaction with peers among U.S. adolescents in the 21st century and links to loneliness. J. Soc. Pers. Relatsh..

[11] Barreto M., Victor C., Hammond C., Eccles A., Richins M.T., Qualter P. (2021). Loneliness around the world: Age, gender, and cultural differences in loneliness. Personal. Individ. Differ..

[12] Hawkley L.C., Buecker S., Kaiser T., Luhmann M. (2022). Loneliness from young adulthood to old age: Explaining age differences in loneliness. Int. J. Behav. Dev..

[13] Jeste D.V., Lee E.E., Cacioppo S. (2020). Battling the Modern Behavioral Epidemic of Loneliness: Suggestions for Research and Interventions. JAMA Psychiatry.

[14] Ernst M., Niederer D., Werner A.M., Czaja S.J., Mikton C., Ong A.D., Rosen T., Brähler E., Beutel M.E. (2022). Loneliness before and during the COVID-19 pandemic: A systematic review with meta-analysis. Am. Psychol..

[15] Berger K., Riedel-Heller S., Pabst A., Rietschel M., Richter D. (2021). Einsamkeit während der ersten Welle der SARS-CoV-2-pandemie–Ergebnisse der NAKO-Gesundheitsstudie. Bundesgesundheitsblatt-Gesundheitsforschung-Gesundheitsschutz.

[16] Buecker S., Horstmann K.T. (2022). Loneliness and social isolation during the COVID-19 pandemic. Eur. Psychol..

[17] Veronese N., Galvano D., D’Antiga F., Vecchiato C., Furegon E., Allocco R., Smith L., Gelmini G., Gareri P., Solmi M. (2021). Interventions for reducing loneliness: An umbrella review of intervention studies. Health Soc. Care Community.

[18] Gu J., Strauss C., Bond R., Cavanagh K. (2015). How do mindfulness-based cognitive therapy and mindfulness-based stress reduction improve mental health and wellbeing? A systematic review and meta-analysis of mediation studies. Clin. Psychol. Rev..

[19] Singer T., Engert V. (2019). It matters what you practice: Differential training effects on subjective experience, behavior, brain and body in the ReSource Project. Curr. Opin. Psychol..

[20] Kok B.E., Singer T. (2017). Effects of Contemplative Dyads on Engagement and Perceived Social Connectedness Over 9 Months of Mental Training: A Randomized Clinical Trial. JAMA Psychiatry.

[21] Kabat-Zinn J. (2003). Mindfulness-based interventions in context: Past, present, and future. Clin. Psychol. Sci. Pract..

[22] Teoh S.L., Letchumanan V., Lee L.-H. (2021). Can Mindfulness Help to Alleviate Loneliness? A Systematic Review and Meta-Analysis. Front. Psychol..

[23] Hickin N., Käll A., Shafran R., Sutcliffe S., Manzotti G., Langan D. (2021). The effectiveness of psychological interventions for loneliness: A systematic review and meta-analysis. Clin. Psychol. Rev..

[24] Lindsay E.K., Young S., Brown K.W., Smyth J.M., Creswell J.D. (2019). Mindfulness training reduces loneliness and increases social contact in a randomized controlled trial. Proc. Natl. Acad. Sci. USA.

[25] Bessaha M.L., Sabbath E.L., Morris Z., Malik S., Scheinfeld L., Saragossi J. (2020). A Systematic Review of Loneliness Interventions Among Non-elderly Adults. Clin. Soc. Work. J..

[26] Richardson L., St Pierre E. (2000). A method of inquiry. Handb. Qual. Res..

[27] Kramer G.P. (1997). Insight Dialogue and Insight Dialogic Inquiry. PhD Thesis.

[28] Singer T., Kok B.E., Bornemann B., Bolz M., Bochow C. (2016). The ReSource Project: Background, Design, Samples, and Measurements.

[29] Cacioppo J.T., Patrick W. (2008). Loneliness: Human Nature and the Need for Social Connection.

[30] Hutcherson C.A., Seppala E.M., Gross J.J. (2008). Loving-kindness meditation increases social connectedness. Emotion.

[31] Russell D., Peplau L.A., Cutrona C.E. (1980). The revised UCLA Loneliness Scale: Concurrent and discriminant validity evidence. J. Personal. Soc. Psychol..

[32] Wei M., Russell D.W., Zakalik R.A. (2005). Adult attachment, social self-efficacy, self-disclosure, loneliness, and subsequent depression for freshman college students: A longitudinal study. J. Couns. Psychol..

[33] Akin A. (2010). Self-compassion and Loneliness. Int. Online J. Educ. Sci..

[34] Zhang N., Fan F., Huang S., Rodriguez M.A. (2018). Mindfulness training for loneliness among Chinese college students: A pilot randomized controlled trial. Int. J. Psychol..

[35] Gierveld J.d.J. (1998). A review of loneliness: Concept and definitions, determinants and consequences. Rev. Clin. Gerontol..

[36] Jazaieri H., Jinpa G.T., McGonigal K., Rosenberg E.L., Finkelstein J., Simon-Thomas E., Cullen M., Doty J.R., Gross J.J., Goldin P.R. (2013). Enhancing Compassion: A Randomized Controlled Trial of a Compassion Cultivation Training Program. J. Happiness Stud..

[37] Godara M., Silveira S., Matthäus H., Heim C., Voelkle M., Hecht M., Binder E.B., Singer T. (2021). Investigating differential effects of socio-emotional and mindfulness-based online interventions on mental health, resilience and social capacities during the COVID-19 pandemic: The study protocol. PLoS ONE.

[38] Bagby R.M., Parker J.D.A., Taylor G.J. (1994). The twenty-item Toronto Alexithymia scale—I. Item selection and cross-validation of the factor structure. Journal of Psychosomatic Research.

[39] Kroenke K., Spitzer R.L., Williams J.B.W. (2001). The PHQ-9: Validity of a brief depression severity measure. Journal of General Internal Medicine.

[40] Spitzer R.L., Kroenke K., Williams J.B.W., Löwe B. (2006). A Brief Measure for Assessing Generalized Anxiety Disorder: The GAD-7. Arch. Intern. Med..

[41] Döring N., Bortz J. (1993). Psychometrische Einsamkeitsforschung: Deutsche Neukonstruktion der UCLA Loneliness Scale. Diagnostica.

[42] Aron A., Aron E.N., Smollan D. (1992). Inclusion of other in the self scale and the structure of interpersonal closeness. J. Personal. Soc. Psychol..

[43] Neff K.D., Tóth-Király I., Knox M.C., Kuchar A., Davidson O. (2021). The development and validation of the state self-compassion scale (long-and short form). Mindfulness.

[44] Carver C.S. (1997). You want to measure coping but your protocol’too long: Consider the brief cope. Int. J. Behav. Med..

[45] Gilbert P., McEwan K., Gibbons L., Chotai S., Duarte J., Matos M. (2012). Fears of compassion and happiness in relation to alexithymia, mindfulness, and self-criticism. Psychol. Psychother. Theory Res. Pract..

[46] Faul F., Erdfelder E., Lang A.-G., Buchner A. (2007). G* Power 3: A flexible statistical power analysis program for the social, behavioral, and biomedical sciences. Behav. Res. Methods.

[47] Bates D., Mächler M., Bolker B., Walker S. (2015). Fitting Linear Mixed-Effects Models Using lme4. J. Stat. Softw..

[48] Hothorn T., Bretz F., Westfall P. (2008). Simultaneous inference in general parametric models. Biom. J. J. Math. Methods Biosci..

[49] R Core Team (2022). R: A Language and Environment for Statistical Computing.

[50] Rosseel Y. (2012). lavaan: An R package for structural equation modeling. J. Stat. Softw..

[51] Cohen J. (1992). A power primer. Psychol. Bull..

[52] Clark-Gordon C.V., Bowman N.D., Goodboy A.K., Wright A. (2019). Anonymity and online self-disclosure: A meta-analysis. Commun. Rep..

